# BROODING MODERATES THE RELATIONSHIP BETWEEN CEREBROVASCULAR BURDEN AND VASCULAR DEPRESSION

**DOI:** 10.1093/geroni/igz038.3216

**Published:** 2019-11-08

**Authors:** Manuel Herrera Legon, Daniel Paulson

**Affiliations:** University of Central Florida, Orlando, Florida, United States

## Abstract

Objective: The vascular depression hypothesis posits that cerebrovascular burden confers risk for late-life depression. Though neuroanatomical correlates of vascular depression (prefrontal white matter hyperintensities) are well established, little is known about cognitive correlates; the identification of which may suggest therapeutic targets. Aims of this study are to examine the hypothesis that the relationship between cerebrovascular burden and depressive symptoms is moderated by brooding, a type of rumination. Method: A sample of 52 community-dwelling, stroke-free, individuals over the age of 70, without history of severe mental illness or dementia completed the Ruminative Responses Scale, and provided self-report (cardiac disease, hypertension, diabetes, high cholesterol) CVB data. The Geriatric Depression Scale was used to assess depressive symptomatology. Results: Results of a bootstrapped model were that self-reported measures of CVB predicted depressive symptomatology. This relationship was significantly moderated by brooding. Among older adults, those who self-reported high CVB and medium to elevated levels of rumination experienced disproportionately more depressive symptomatology. Conclusions: These findings suggest that brooding rumination may be one correlate of the vascular depression syndrome. Future research should examine neuroanatomical correlates of rumination among older adults, and further explore brooding as a therapeutic target for those with late-life depression.

